# Direct reprogramming into interneurons: potential for brain repair

**DOI:** 10.1007/s00018-019-03193-3

**Published:** 2019-06-27

**Authors:** Maria Pereira, Marcella Birtele, Daniella Rylander Ottosson

**Affiliations:** Department of Experimental Medical Science and Lund Stem Cell Center BMC, Lund University, 22141 Lund, Sweden

**Keywords:** Transdifferentiation, Viral injections, Neuronal conversion, Intracerebral injections, Mice, Cell therapy, Parvalbumin, Neurodegenerative diseases, Neuropsychiatric disorders, Dopamine, iPSC, ESCs, iNs

## Abstract

The brain tissue has only a limited capacity for generating new neurons. Therefore, to treat neurological diseases, there is a need of other cell sources for brain repair. Different sources of cells have been subject of intense research over the years, including cells from primary tissue, stem cell-derived cells and reprogrammed cells. As an alternative, direct reprogramming of resident brain cells into neurons is a recent approach that could provide an attractive method for treating brain injuries or diseases as it uses the patient’s own cells for generating novel neurons inside the brain. In vivo reprogramming is still in its early stages but holds great promise as an option for cell therapy. To date, both inhibitory and excitatory neurons have been obtained via in vivo reprogramming, but the precise phenotype or functionality of these cells has not been analysed in detail in most of the studies. Recent data shows that in vivo reprogrammed neurons are able to functionally mature and integrate into the existing brain circuitry, and compose interneuron phenotypes that seem to correlate to their endogenous counterparts. Interneurons are of particular importance as they are essential in physiological brain function and when disturbed lead to several neurological disorders. In this review, we describe a comprehensive overview of the existing studies involving brain repair, including in vivo reprogramming, with a focus on interneurons, along with an overview on current efforts to generate interneurons for cell therapy for a number of neurological diseases.

## Cell transplantation and brain repair

Efforts to replace damaged neurons in the brain through transplantation of various cell sources have been carried out over decades for a number of neurological diseases. First attempts date back to the 80’s, when Swedish scientists’ research led to a clinical trial, in which midbrain-derived fetal cells were transplanted into the brain of Parkinson’s disease (PD) patients. The cells survived transplantation and induced symptomatic relief in some patients, integrating in the host brain and re-innervating the striatum, where they released the neurotransmitter dopamine ([1, 2]; reviewed in [3]). Fetal neuronal transplants from whole ganglionic eminences have also been attempted to treat patients with Huntington’s disease (HD), providing several years of improvement and stability, although not a cure to the disease [4]. Despite the potential for brain repair of fetal cells, a limited availability of tissue and important ethical concerns, limited the use of therapies involving these cells as they cannot be available for a large number of patients. There was hence a need for developing other cell sources that could be used for this purpose. Beside the cell of origin, the host environment needs to be considered for transplant integration [2, 5]. In this context, GABAergic interneurons (INs) that derive from medial ganglionic eminence (MGE) showed the capacity to disperse and integrate into neural circuits in the postnatal brain (reviewed in [6, 7]). Recent advances in cellular biology and improvement of culture methodologies have led to the development of protocols that allow the use of embryonic stem cells (ESCs), pluripotent cells that can be obtained from human blastocysts [8], expanded in vitro and differentiated into specific types of neurons or neural progenitors for cell replacement. hESCs have been successfully used as a source of neurons that show molecular, biochemical and functional traits of *bonafide* dopaminergic neurons (DA), generated using extrinsic patterning cues that mimic fetal brain development [9, 10]. Also layer-specific cortical neurons [11, 12], GABAergic and serotoninergic neurons [13], motor neurons [14, 15], peripheral neurons [16, 17] and neural progenitor cells have been generated in vitro from hESCs [18, 19]. Reports of human stem cell differentiation into MGE-derived INs, such as Parvalbumin (PV)- and Somatostatin (SST)-positive cells, haven’t always shown high efficacy, even when long-term co-culture was used [20, 21].  However, differentiation into INs has seen significant progress, with more efficient differentiation into subtype-specific groups of INs or forebrain-specific GABAergic INs [22–24].

Limitations associated with the use of ESCs for neuron derivation are related with the pluripotency of the starting cell. While this does not preclude their use in the clinic, extensive (and expensive) preclinical testing is required prior to use. Additionally, there are ethical considerations as well as issues related to high cost, patentability and commercialization of products derived from human embryos that could hamper the development of such therapies [25, 26].

In 2006, Takahashi and Yamanaka identified four factors (*Oct3/4, Klf4, Sox2* and *Myc*) that were sufficient to directly reprogram mouse adult somatic cells into an induced pluripotent stem cell state, generating the so-called induced pluripotent stem cells (iPSCs) [27]. This cell type could allow for patient-specific autologous grafts, lowering the risk of graft rejection and therefore circumventing the need for immunosuppression. Their potential use in the clinic could also raise less ethical concerns due to the autologous origin of the cells. Human iPSCs can be differentiated using similar protocols as the ones used for ESC differentiation, and generate subtype-specific neurons that survive transplantation [21, 28–30]. In addition to the cell therapy potential, iPSCs offer an unlimited source of patient-derived cells, which in principle can be differentiated into disease-relevant somatic cell types to create in vitro models of the disorder of interest [31].

Despite their potential for personalized treatments, iPSCs’ use also raises concerns regarding the feasibility as a therapy for a large population of patients, as there would be important technical, regulatory and financial drawbacks, mostly related to their autologous origin. If using iPSCs from autologous sources, a more difficult standardization of manufacturing processes and consequently higher costs, could arise from such therapy [32].

**Fig. 1:**
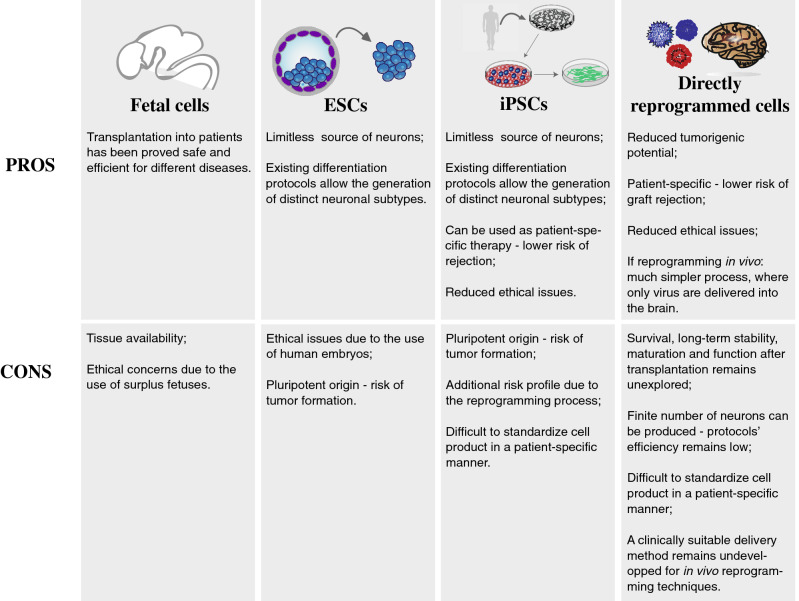
Pros and Cons of different cell sources considered for cell replacement therapies

Alternatives to transplantation of these sources involve the use of somatic cells that can be directly reprogrammed into fully functional mature neurons of specific subtypes without passing through the stage of pluripotency. In Fig. 1, we present an overview of the different cell sources that are considered for cell replacement in the brain and the respective pros and cons.

## Direct neuronal reprogramming

### In vitro reprogramming

In recent years and following the finding that somatic cells can be reprogrammed into pluripotency, many labs have also succeeded in directly switching the identity of one cell type to another, without going through a pluripotent state. By expression of appropriate transcriptional factors, with or without the assistance of specific environmental signals, fibroblasts were used for cellular reprogramming into cardiomyocytes, blood progenitor cells, hepatocytes, epiblast stem cells and neural progenitors [33–40], showing that adult somatic cells can be reprogrammed not only to pluripotency, but also into distantly related cell types.

In 2010, researchers showed that by overexpressing as few as three genes, *Ascl1*, *Brn2,* and *Myt1L* (ABM) in mouse embryonic and perinatal skin fibroblasts, these cells could be reprogrammed into neurons, termed *induced neurons *[41]. The same factors were also shown to convert human fibroblasts alone, or in combination with *NeuroD1* [42, 43]. This so-called direct reprogramming into neurons has today developed into a likely approach to obtain functional and subtype-specific neuronal cells that in turn might be used to replace those lost by insults such as in PD, spinal cord injury or psychiatric disorders [44, 45]. *Induced neurons* have a reduced risk of tumorigenic potential due to their non-pluripotent origin and have appealing advantages such as the fact that neurons can be generated from relatively easily obtainable cells like fibroblasts, the significant reduction in ethical concerns due to the autologous origin of the cells, and the lower risk of graft rejection. Besides that, they offer a faster and less labour-intensive option than that of iPSC.

Cellular reprogramming brought new insights into the neuroregenerative medicine field and proposed an appealing strategy to generate neurons of different subtypes. Their use as alternatives for cell therapy has been largely explored in the last decade. With the use of pro-neural and cell-type-specific transcription factors (TFs), as well as micro-RNAs and small molecules, several groups have shown that mouse and human fibroblasts and astrocytes can be reprogrammed into different types of neurons including glutamatergic, GABAergic, motor, sensory and DA neurons [44, 46–53], among others. *Induced neurons* have been generated in vitro and transplanted, showing survival and functional integration in the host brain [44, 47, 54–56].

In vitro reprogramming techniques have also been used to generate GABAergic telencephalic neurons and GABAergic INs. Colasante et al. have shown that both mouse and human fibroblasts and iPSCs can be converted into cortical GABAergic INs upon transduction with a viral cocktail containing important factors for induction of a GABAergic IN fate, such as *Ascl1*, *Dlx5* and *Lhx6*, as well as genes expressed during early forebrain development such as *FoxG1* and *Sox2* [57]. These GABAergic INs were transplanted into the mouse brain and showed to functionally integrate in the host neuronal networks, release GABA and inhibit the surrounding excitatory neurons in the hippocampus. A great part of the GABAergic neurons also showed PV protein and gene expression. Similarly, another group has used in vitro reprogramming to obtain subtype-specific INs only with the aid of one reprogramming factor *Ascl1*, and upon treatment of the cultures with Forskolin, which were able to induce 80% of PV-expressing INs from mouse fibroblasts [58].

Directly reprogrammed neurons can, similarly to iPSCs, offer a cell-based and patient-specific source of neurons for disease modelling, while also involving much less ethical concerns compared to hESCs.

For cell repair strategies, reprogramming techniques can be used both for generating neurons in vitro that can then be used for transplantation [42, 47, 50, 56] or to reprogram resident glial cells of the brain into specific types of neurons in situ [59–62]. Thereby resident parenchymal cells could be converted into cell types that are lost due to disease by a process called in vivo reprogramming. This technique has also been used to reprogram cells in tissues other than the brain [63–65]. A more detailed overview of the current status of in vivo reprogramming in the central nervous system (CNS) will be done in the following sections.

### In vivo reprogramming

In vivo reprogramming is still in its early stages but could develop into an option for cell therapy. To date, experiments involving this technique have been performed either by injecting reprogramming factors into the brain, which will then drive reprogramming of resident glia into neurons, or by transplanting cells that were transduced in vitro with reprogramming factors that can then be activated in vivo, post-transplantation. The first approach is thus far performed in mice resident glia and the latter, can involve the use of human cells transplanted to the rodent brain. A large number of reports indicating the feasibility of this technique have been published, and results indicate that different types of neurons can be generated in vivo. The large majority of obtained neurons are either GABAergic or Glutamatergic [45, 59, 60, 66, 67], but also other subtype-specific neurons have been generated such as DA neurons [67, 68] or INs [61, 69] (see Table 1). Thus far, in vivo neuronal reprogramming has been established in different regions of the brain such as the cortex, spinal cord, striatum and the midbrain [45, 61, 70]. Moreover, the reprogramming can take place both in the intact and in lesioned CNS [45, 59, 61, 71] showing its potential for application in a clinical context.

**Table 1:** A summary of published studies involving in vivo direct reprogramming of glial cells into neurons

	Species	Starting cell	Factor combination	Vector system	Animal model	Resulting cell type	Region
Buffo et al. [85]	Mouse	Proliferating glia	Pax6/dominant negative	RV	SW	Transient neurons	Cortex
Torper et al. [62]	Rat	Human astrocytes and fibroblasts	ABM and ABM+4F	Dox-inducible LVs	6-OHDA	Dopaminergic neurons	Str and Hpc
	Mouse	Resident astrocytes	ABM	Cre-inducible LVs	–	–	Str
Niu et al. [69]	Mouse	Astrocytes	Sox2	LVs/cell type specific promoter	Young, adult and aged mice	Neuroblasts	Str
Grande et al. [59]	Rat	Proliferating cells	Ngn2	RVs	SW and ischaemia	GABAergic and Glutamatergic neurons	Str
Su et al. [45]	Mouse	Astrocytes	Sox2	LVs/cell type specific promoter	SCI	Neuroblasts (GABAergic interneurons and glutamatergic neurons, after VPA)	Spinal cord
Guo et al. [66]	Mouse	Astrocytes and NG2 glia	NeuroD1	RVs/cell type specific promoter	SW and AD model	Glutamatergic (Astrocyte-derived) , Glutamatergic and GABAergic (NG2-derived)	Cortex
Magnusson et al. [77]	Mouse	Astrocytes	Block notch signalling	Transgenic mice/AAVs	Intact and stroke	Neurons	Str
Heinrich et al. [100]	Mouse	NG2 glia	Sox2	RVs	SW	Neurons	Cortex
Niu et al. [98]	Mouse	Astrocytes	Sox2	LVs/cell type specific promoter	–	Neural progenitors (Calretinin+ interneurons, after VPA)	Str
Torper et al. [67]	Mouse	Astrocytes and NG2 glia	ALN	Cre-inducible AAVs	–	GABAergic and Glutamatergic neurons	Str
Liu et al. [60]	Mouse	Astrocytes	Ascl1	AAVs/cell type specific promoter	–	GABAergic and Glutamatergic neurons	Dorsal midbrain, Str and cortex
Di Val Cervo et al. [71]	Mouse	Astrocytes	NeAL218	Tet-regulated LVs/GFAP-tTA mice	6-OHDA	TH+ neurons	Str
Weinberg et al. [70]	Rat	Oligodendrocytes	PTB inhibitor/ miRNA	Oligodendrocyte-specific AAVs	–	Striatal neurons	Str
Brulet et al. [99]	Mouse	Non-reactive astrocytes	NeuroD1	Systemic injection/AAV9	–	–	Str and cortex
Pereira et al. [61]	Mouse	NG2 glia	ALN, NgLN, ANgN, NgND1 AFLE	Cre-inducible AAVs	Intact vs 6-OHDA	Parvalbumin+ interneurons	Midbrain and Str
Niu et al. [68]	Mouse	Striatal MSNs	Sox2, Nurr1, Lmx1A, FoxA2, Valproic acid	LVs/cell type specific promoter or hPGK	–	TH+ neurons	Str
Matsuda et al. [83]	Mouse	Microglia	NeuroD1	LVs/cell type specific promoter	–	DARPP32+ striatal projection neurons	Str

*ABM* Ascl1, Brn2, Myt1L, *ABM + 2F* ABM + Lmx1a, Lmx1b, *ABM + 4F* ABM + Lmx1a, Lmx1b, FoxA2, Otx2, *NeAL218* NeuroD1, Ascl1, Lmx1a, miRNA218, *ALN Ascl1*, *Lmx1a*, *Nurr1*, *NgLN Neurogenin2*, *Lmx1a*, *Nurr1*, *ANgN Ascl1*, *Neurogenin2*, *Nurr1*, *NgND1 Neurogenin2*, *Nurr1*, *NeuroD1*, *AFLE Ascl1*, *FoxA2*, *Lmx1a*, *En1*, *LV* lentivirus, *RV* retrovirus; *AAV* adeno-associated virus; *SC* spinal cord, *SCI* spinal cord injury, *SW* stab-wound, *Str* striatum, *Hpc* hippocampus, *MSNs* medium-spiny neurons

To fully understand the process and its limitations, aspects such as (1) the cell of origin, (2) genes used for reprogramming, (3) chosen delivery systems, and (4) the region where reprogramming occurs and what effect this might have on functionality, need to be explored. In the following sections, a discussion on these aspects as well as an overview on the existing in vivo studies where subtype-specific neurons were generated will be made.

### Cell of origin

The first important aspect to consider for in vivo reprogramming studies is the identification of the cell type that is most suited to undergo conversion into the desired cell type, and that is available at the right place. It has been hypothesised that the selection of the source cell type will impose a specific molecular context, defined by its gene expression profile and epigenetic signature, in which reprogramming factors will have to operate [72]. Based on this, different resident cell types of the brain have been considered for in vivo reprogramming, including glial cells such as astrocytes and oligodendrocyte precursor cells, also known as NG2 glia. These cells play multiple roles in helping to alleviate neurological defects after brain injury, by promoting axonal and neuronal function and thereby fostering neuronal survival [73]. Astrocytes could be advantageous to use for cell reprogramming in vivo due to their ubiquitous distribution throughout the CNS in large amounts and the fact that they are associated with a high plasticity [74]. A recent paper points to a heterogenous susceptibility for reprogramming by astrocytes derived from different parts of the brain [75]. Furthermore it has been demonstrated that astrocytes can be triggered into a tripotent differentiating and self-renewing state after an injury, which could potentially create the appropriate context for reprogramming into neurons [76]. Remarkably, recent data demonstrate that astrocytes seem to entail a latent neurogenic program that can be initiated upon injury [77]. The fact that striatal astrocytes are able to produce neuroblasts in models of Stroke or Huntington’s disease sheds new light on the neurogenic potential of the adult brain and raises questions on whether we can promote renewal of striatal neurons for therapeutic approaches. Further debate on adult neurogenesis has been supported by a recent paper that demonstrates persistence in neurogenesis during both physiological and pathological aging in humans [78] and this in turn points to a potentially relevant mechanism underlying Alzheimer’s disease symptoms.

The extensive self-renewal capability of NG2 glia [79] also makes them interesting candidates for in vivo reprogramming, as the potential risks in disturbing homeostasis can be avoided. In addition, NG2 glia receives direct input from neurons [80], so the necessary machinery to form post-synaptic compartments is already in place, potentially facilitating the synaptic integration of reprogrammed, NG2-derived neurons. Also, these glial cells may hold the key to overcome another major problem faced in attempts to replace degenerated neurons, namely, the long-term survival of the newly regenerated neurons [73].

Besides astrocytes and NG2-glia, other cells have been implicated for reprogramming such as specific sets of pericytes, a cell type that lines the inside of capillaries in the CNS [81] or microglial cells that have the ability to migrate to the lesion site. The migration of monocyte-derived macrophages to the lesion area from blood [82] has attracted attention due to the potential as therapeutic delivery strategy for reprogramming factors. For this, monocytes could be genetically modified to express inducible reprogramming factors and administered by systemic injection into the subject, being reprogrammed into neurons when reaching the CNS. In a recent publication, the feasibility of microglia-to-neuron in vivo reprograming in the striatum of the adult mouse has been demonstrated through the overexpression of a single transcription factor [83].

Upon neurodegeneration or brain injury, glial cells undergo important morphological, functional and molecular changes, which initially promote tissue repair, but later can contribute to adverse effects for neuronal survival as well as neurite and synapse formation. A specific and timed reprogramming of these cells into neurons could provide a new therapeutic approach for brain repair. Ideally, one would want to selectively convert scar-forming glial cells into neurons, while avoiding an effect on the populations of glia that are crucial for effective wound healing processes [84]. Other cell sources involved in glial scar formation also possess some potential for in vivo reprogramming. By forcing the reactive glial cells to express specific TFs such as *Pax6* together with a dominant negative form of *Olig2*, Buffo et al. showed that transient immature neurons can be formed in the mouse brain after a stab-wound-induced cortical brain injury [85].

Taken together, new insights into glial cell diversity have opened new avenues towards best utilizing the CNS-resident cells for repair purposes. Yet, much still remains to be understood about this diversity to be putting it at its best use.

In contrast to glial cells that can become reactive and proliferate under certain conditions, post-mitotic neurons do not normally change their identity for the lifespan of the organism, especially not during adulthood. Nevertheless, a recent paper identified striatal neurons as the cell source for neuronal reprogramming in the adult mouse brain [68]. In this paper, in vivo reprogramming was promoted in the striatum by the transcription factors *Sox2*, *Nurr1*, *Lmx1a*, and *FoxA2* along with the chemical factor valproic acid. Immunohistochemistry and genetic lineage tracing revealed that in the origin of induced DA neurons were the striatal neurons. This data indicates that neurons can be redirected to other phenotypes also in the adult brain.

### Reprogramming process, genes, and delivery

It is still largely undefined how an individual cell transits from its original identity to a neuronal fate during cellular reprogramming. Even so, recently developed techniques are now unravelling this process bit-by-bit. By analysing transcriptomes of cell populations and single cells at different time points after viral transduction, two studies have contributed importantly to the field by showing that the process of direct reprogramming might involve an immature intermediate state and is not purely a transition between fully differentiated stages [86, 87]. Overexpression of the proneuronal pioneer factor *Ascl1* in mouse embryonic fibroblasts led to two gene regulatory events during reprogramming starting with an initiation stage where the cells exit the cell cycle and cytoskeletal reorganization, followed by a maturation stage, where genes involved in synaptic maturation are turned on [87]. Human brain pericytes have been shown to transiently express neural stem cell-like genes and then to enter a bifurcation in lineage differentiation into distinct excitatory or inhibitory pathways [86]. Interestingly the two groups of starting populations described in this publication, differed markedly in their response to the reprogramming genes *Ascl1* and *Sox2*, demonstrating that the reprogramming success critically depends on cellular context [86].

That might explain why several factors that have been shown to convert astrocytes into neurons in vitro [88, 89], yet fail to do so in vivo [59]. Some of the genes that have been effective at reprogramming somatic cells into DA neurons in vitro, give rise to INs instead when used for in situ reprogramming of resident glia in the striatum [61]. The fact that in vivo reprogramming studies have not yet convincingly and reproducibly shown the generation of a DA neuronal subtype in vivo, raises the question of whether the activation cascades necessary for the formation of DA cells in situ are somehow dependent of the ones involved in the formation of INs, or whether the IN subtype is the default subtype formed when these genes are introduced in the brain. These different outcomes could also reflect differences between cellular identity of resident glial populations and the starting population in the in vitro studies.

It has been accepted in the field of cellular reprogramming that genes that are important during development in the formation and specification of a specific neuronal subtype, often are effective at reprogramming an adult somatic cell into that neuronal subtype as well [90]. Hence, understanding how these processes work is of great importance and provides valuable information for reprogramming studies. As an example and in support of this, many of the genes identified during midbrain DA neuron formation in vivo have already been used to reprogram somatic cells into induced DA neurons in vitro [42, 44, 60, 91–93].

GABAergic IN fates arise from an important interplay of genes, expressed in early progenitor stages. Genes like *Ascl1*, *Dlx5* and *Lhx6* are considered inducers of a GABAergic IN fate [94–97]. It has been shown that these genes, together with *Sox2* and *FoxG1*, are able to induce an IN fate when fibroblasts are transduced in vitro, and that *Ascl1*, *Dlx5*, and *Lhx6* have a more direct cooperation in activating the molecular machinery responsible for GABAergic specification [57]. In support of this hypothesis, the authors of this study have shown that silencing of either *FoxG1* or *Sox2* is sufficient to block, in a cell-autonomous manner, the ability of *Ascl1* to induce a GABAergic neuronal fate in cortical progenitor cells. In two separate studies, *Sox2* has been shown to be sufficient to reprogram astrocytes into INs in the mouse CNS [45, 98] (see Table 1).

When performing in vivo reprogramming in the brain, one needs to account for the correct targeting of specific cell populations. Viral vectors are commonly used to deliver genetic material into the brain cells. To target proliferating cells, retroviral vectors (RVs) can be used since these vectors specifically target dividing cells [59, 66]. Alternatively, lentivirus (LVs) and adeno-associated (AAV) vectors can be used, and these have the advantage of infecting both dividing and non-dividing cells of the brain [59, 66, 99]. To target specific cell populations in the brain, subtype-specific promoters are used for the reprogramming genes in viral constructs [66, 98].

Cre-inducible LV or AAV vectors have been used to reprogram resident glial cells into neurons in the mouse brain, and using a neuron-specific synapsin-driven FLEX reporter, overexpressed in these in vivo reprogrammed neurons, the number of newly formed mature neurons with a glial origin could be easily determined, providing for an immediate method to identify and characterize those neurons [61, 67]. On the other hand, with these viruses it remains difficult to determine the exact number of targeted cells in vivo as only the converted cells are identified. Other viral systems, e.g. encoding a fluorescent reporter that is not expressed under neuron-specific promoter works better for identification and quantification of transduced cells [100, 48].

Finding an appropriate and safe system for gene delivery in humans is also under consideration in the field. Certain serotypes of AAVs have been reported to successfully cross the blood–brain barrier, opening up the possibility to transduce cells in the brain through systemic injection (reviewed in [84]). This could avoid secondary damage due to invasive cerebral injections, and allow transductions in larger areas, required in later stages of neurodegenerative diseases.

### Region/subtype and functionality

For in vivo reprogramming to become an attractive possibility for future brain repair, we need to control the subtype identity and functionality of the generated neurons more effectively. Indeed for in vivo reprogramming, environmental regional differences have been observed in several parts of the brain. While the transcription factor *Ngn2* alone can generate GABAergic neurons in the striatum, the same factor induced glutamatergic cells in the neocortex [59] (see Table 1). Besides, brain insults seem to differentially modulate the generation of new neurons in vivo [59, 100].

**Fig. 2:**
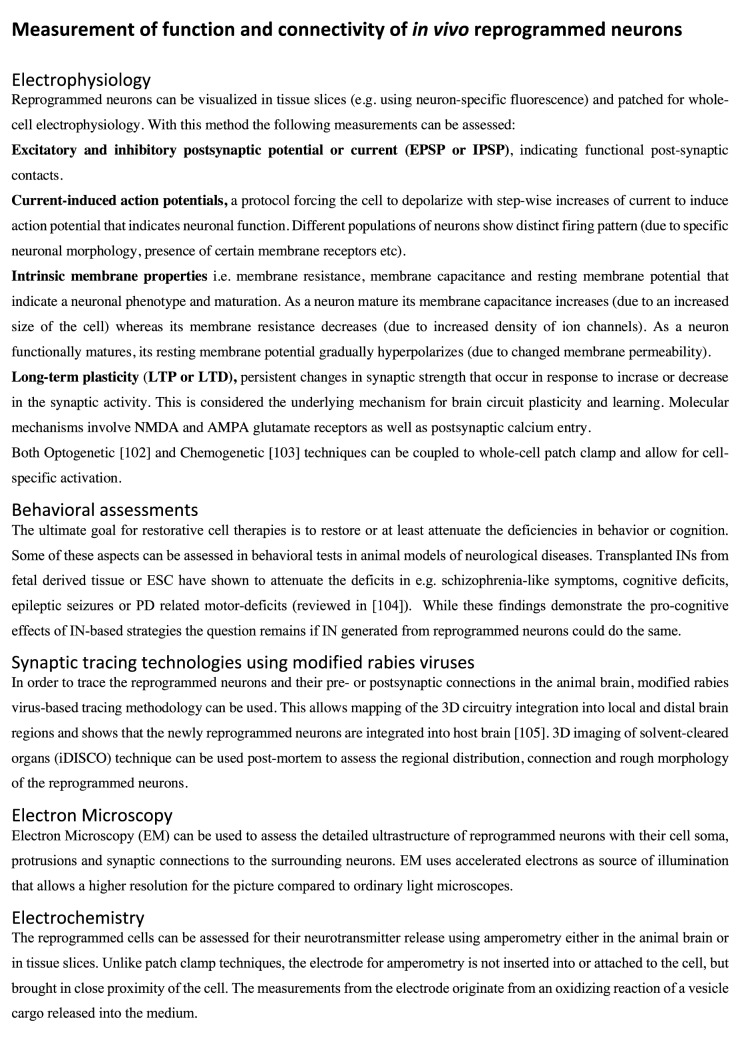
Methods for functional assessment of reprogrammed neurons

It is important to assess the final outcome of direct reprogramming in terms of function and connectivity of reprogrammed cells in the adult brain. The formation of synapses and neuronal function, with ability to electrically communicate, is necessary for a neuron to exert any behavioural effect including attenuating the disease symptoms [101]. The first evidence that reprogrammed neurons functionally mature in the brain was provided by [66]. Patch-clamp electrophysiological recordings (see Fig. 2, [102–105]) of cortical tissue slices revealed a capability of both evoked and spontaneous action potentials for the new neurons obtained in *NeuroD1*-reprogrammed astrocytes around 4 weeks after transduction. Another group used *Sox2* to reprogram NG2 glia into neuroblasts, which further matured into neurons, capable of firing action potentials and exhibiting post-synaptic currents in vivo [100].

NG2-glia-derived reprogrammed neurons in the intact striatum have been further studied using the same technique, to demonstrate a gradual maturation in function with capacity for evoked repetitive action potentials and post-synaptic currents from 5 to 12 weeks after virus injection [61]. It was here shown that the functional properties of the reprogrammed neurons correlated to maturation over time, and by 12 weeks after viral injection, the reprogrammed cells show molecular and functional properties of endogenous striatal neurons. Moreover, the membrane-related intrinsic properties also indicated a gradual maturation, i.e., membrane capacitance increased, while the membrane resistance decreased, and the resting-membrane potential became more hyperpolarized (see Fig. 2). Neurons reprogrammed from NG2-glia further received synaptic input from local host neurons as shown by monosynaptic tracing [67] (Fig. 2).

### In vivo reprogramming into subtype-specific neurons

After the first evidence that in vivo neuronal reprogramming of resident glial cells could occur in situ [85], research groups further explored this approach with the aim of using it for CNS repair. Torper et al. introduced factors in resident striatal astrocytes that were shown to reprogram somatic cells into DA neurons in vitro (*Ascl1, Brn2* and *Myt1L* [41]), and stably converted these into NeuN-expressing neurons in the adult mouse brain [62]. In the same year, another group reported that LV-mediated overexpression of *Sox2,* driven by a GFAP-specific promoter could reprogram adult striatal astrocytes into neuroblasts that could later form neurons upon exposure to noggin and BDNF, or upon treatment with a histone deacetylase inhibitor [69]. Further reports over the years have shown reprogramming of different cell types [59, 66, 98, 100] into neuroblasts or neurons in vivo, in different regions of the brain and spinal cord [45, 60], through the use of distinct factor combinations and delivery methods [67, 100] (see Table 1). Among these, two studies have shown the generation of DA neurons in the striatum of intact or lesioned brains targeting either glia [71] or striatal neurons [68].

Recently, even the epigenetic changes that occur during reprogramming have been described as the underlying mechanism during transdifferentiation [106, 107]. Matsuda and co-authors have been pioneers exploring in detail the epigenetic regulation of the neuronal factor* NeuroD1* on the in vivo reprogrammed neurons from microglia. The authors point to a global epigenetic remodelling done by *NeuroD1*, starting with an initial onset of a neuronal program and consecutive downregulation of microglial genes [83].

Thus far, functional assessment and protein or gene expression have been sparse and the different neuronal subtypes generated in the animal brain have mainly been characterised as either GABAergic or Glutamatergic [59, 67]. Our lab has been one of the few that further evaluated the subtype-specificity of in vivo reprogrammed neurons. Using genome analysis, protein-expression and electrophysiology, we could characterize the reprogrammed neurons in the striatum and conclude that a big portion of the neurons (40%) showed properties similar to fast-spiking GABAergic INs expressing PV [61], a cell type that usually accounts only for 1% of all striatal neurons and that plays a highly interesting role in striatal function. A minority of the neurons expressed markers and showed functionality traits similar to other types of striatal INs, whereas very few cells showed properties of neurons more abundantly found in the striatum like medium-spiny neurons [61]. GABAergic INs have previously been generated in vivo both in the latent state and after a trauma such as excitotoxic lesion [108] or stroke (without addition of any reprogramming factors) or with Notch signalling inhibition [77] or with *Sox2* induction [45, 98]. The studies from Su et al. and Niu et al. 2015 have further showed neuronal reprogramming into GABAergic INs in the spinal cord and striatum; however, under different conditions and with unreported proportion of cells (see Table 1). In Niu et al. 2015, the reprogrammed astrocytes in the striatum showed properties of calretinin INs [98], whereas Su et al. 2014 reprogrammed astrocytes into GAD65-expressing INs in the spinal cord [45].

## Interneurons: the re-shapers of neural networks

INs have an essential role in balancing and coordinating different networks in the nervous system [109]. In fact, INs populate the spinal cord and the brain, both cortically and subcortically, with a great variability in cell types. The identification of different types of INs done so far is based on different aspects: firing properties, immunohistochemical profile and gene expression. For the striatal INs, the electrophysiological profile allows distinguishing between the following groups: fast-spiking INs (FSI), characterised by low input resistance and a fast firing pattern; low threshold INs (LTI), represented by a high input resistance and a sustained plateau potential present after current injections; and tonic active cholinergic INs (TANs), which have an hyperpolarization-activated current and long spike after-hyperpolarizations [110]. Another criterion used to classify the IN populations is their immunohistochemical profile, as they can express PV, SST, neuropeptide Y (NPY), nitric oxide synthase (NOS), and calretinin [110, 111]. GABAergic INs besides GABA, express the Ca^2+^ -binding protein PV, the neuropeptide SST, and the ionotropic serotonin receptor (5HT3a), (reviewed in [112–114]) whereas excitatory INs express gastrin-releasing peptide or substance P [115]. Although different INs are found in distinct parts of the brain, the identification of the exact type of IN is not always easy to make. More knowledge about the existing subtypes would be important as it could bring and possibly influence future therapeutic approaches [116].

INs work as a buffer system of the excitatory signals avoiding runaway excitation [117]. This role is in line with their sparse localisation amongst other cells [118] and their lack of major distant projections [119] for the majority of INs [117]. INs function not merely as guards of excitatory networks with feed-back inhibition, but they also contribute to the general activity with motif-like feed-forward inhibition which allows activity signal synchronisation and long-term alteration of cellular excitability [120]. Another characteristic of INs is represented by their ability for electrical coupling between different regions of the brain that allows the regulation of chemical synapse development and circuits’ formation in the neocortex [121–123]. In addition to principal cells, neocortical GABAergic INs are also known to target other INs, giving rise to disinhibitory effect that is found in different regions of the brain [124, 125]. At last, INs can drive cortical plasticity and contribute to the reshaping of neural networks [126, 127]. In this sense, they are paramount in balancing excitatory and inhibitory signalling, a balance that when disturbed is correlated to several neurological conditions and psychiatric disorders such as autism, schizophrenia, and intellectual disabilities [128].

## Interneuron dysfunction in psychiatric disorders and cell reprogramming

Interneuropathies constitute a wide range of neurological disorders that directly result from IN dysfunctions [129]. Whether they are caused by a reduction in IN number or more specific deficits in the firing properties of individual neurons, these syndromes all share impaired GABAergic transmission [104]. As GABAergic INs are the main cellular elements in controlling excitability in the brain, severe GABAergic deficits can cause a pathological hyperexcitability. In line with this, many of the genes that are linked to epilepsy are involved in the regulation of IN development and function. Recent data also point to that subtle perturbations in the excitatory–inhibitory balance existing in other psychiatric conditions and neurological disorders [128] such as Alzheimer’s disease [130], chronic pain, dystonia [131], PD, schizophrenia and anxiety (reviewed in [5]). For example, several models of schizophrenia have shown an alteration in the population of PV-expressing INs, with changes in their number and positioning [132]. In autism, a reduced number of PV-expressing INs have been reported, along with a reduced mRNA level of GAD67 and the GABA membrane transporter 1 (GAT1) [133]. Reduced striatal PV expression has further been shown in a dystonia animal model where a delayed IN maturation was speculated to be involved in the pathophysiology [131]. There are also disturbances in biochemical markers in the PV INs such as voltage-gated potassium or sodium channels that have been associated to schizophrenia or AD [130, 134]. The selective reduction in neuronal activity of the PV INs is hypothesised to lead to an attenuation of gamma oscillations that underlie autism, schizophrenia and other diseases [135, 136]. A feature that is common to a broad spectrum of neuropsychiatric disorders is repetitive behaviours such as the ones featured in Tourette’s syndrome. Here, compelling evidence implicates a decrease in striatal PV INs in the emergence of repetitive behaviour both in animal models [137] and in patients [138, 139]. In a DAT-overexpressing rat model that displays behavioural abnormalities, the repetitive behaviour has been linked to decreased number of striatal PV INs and increased c-fos levels in cortical areas [140]. Among other animal models, the mutant mouse Disrupted-in schizophrenia 1 (human full length DISC1 overexpression) gene has shown to lead to neurodevelopmental changes consistent with the proposed neurodevelopmental origin of schizophrenia, i.e., reduction of PV INs in cortex and striatum and impaired migration of INs from ganglionic eminence [141, 142].

## Future prospects and conclusions

Given the involvement of INs in neuropsychiatric and other neurological diseases, the question that naturally occurs is whether we could attenuate any of the neurological or psychiatric symptoms by replacing INs. Recent studies suggest the possibility of tackling several of the above-mentioned diseases with transplants of INs derived from fetal tissue or MGE to the spinal cord to treat SCI, to the striatum for PD, hippocampus for epilepsy and cerebral cortex for psychiatric diseases [5, 66, 104]. Yet, IN replacement for these diseases has largely been neglected in preclinical research. To move this research forward, it is imperative to develop tools and technologies that address the most urgent questions relating to the therapeutic potency of INs, such as their genetic profile and electrophysiological maturation, and synaptic connectivity in the brain (see Fig. 2). Moreover, it is imperative to generate subtype-specific neurons that are matching their endogenous counterparts and to transplant or in vivo reprogram these in the correct target brain region. The ability to generate subtype-specific INs via cell reprogramming opens up for new and safer sources of clinically relevant neurons in the future, as the novel cells are not formed via a proliferative cell intermediate. Notably, this direct reprogramming process is potentially much faster than generating iPSCs or differentiating ESCs into the target cell types, which could take months. Moreover, while iPSCs do not retain their age-related signatures, the directly reprogrammed neurons still display their age-related transcriptional profiles and associated nuclear transport [143]. This allows us to model age-associated processes in vitro, an aspect that is particularly valuable when studying neurodegenerative diseases. In line with this, reprogrammed cells could provide an in vitro platform for drug discovery, toxicology studies and gene therapy testing. Using this approach, it has been shown that overexpression of a gene linked to schizophrenia and bipolar disorder in reprogrammed GABAergic neurons, led to reduced inhibitory synaptic transmission [144].

Expandable cell sources like embryonic stem cells, neural stem cells and more recently described iPSCs, are all under investigation for brain repair. Even though recent progress has been made in differentiating pluripotent stem cells into the appropriate neuronal subtype, fetal transplants are still the gold standard as these are the best specified to differentiate into the appropriate type of neurons. Nevertheless, attempts to use endogenous cell sources are very attractive as they would eliminate the dependence of an exogenous cell source, removing ethical concerns in terms of donor cell origin, difficulties in meeting GMP requirements and logistical issues that are associated with extrinsic cell sources. Therefore, it could be worth improving and developing in vivo reprogramming technique, e.g. by defining the most appropriate cells to target or to define what region-specific features are present in the environment where reprogramming occurs, and how they may affect the final cell identity, functionality and integration. Furthermore, to expect functional recovery, the reprogrammed neurons have to integrate and function as their endogenous counterparts. The most important task though, remains to generate an adequate number of subtype-specific neurons into the brain that can restore network alterations in a specific disease. There are still challenges to achieve these goals but the ability to generate subtype-specific neurons via in vivo reprogramming could open up for new and safer sources of clinically relevant neurons in the future.
